# CLOUD – CeMM library of unique drugs

**DOI:** 10.1186/1758-2946-4-S1-P23

**Published:** 2012-05-01

**Authors:** Patrick Markt, Gerhard Dürnberger, Jacques Colinge, Stefan Kubicek

**Affiliations:** 1 CeMM Research Center for Molecular Medicine, Lazarettgasse 14/AKH BT 25.3, 1090 Vienna, Austria

## 

Approved drugs serve as functional probes for analyzing disease-relevant biochemical pathways or are screened against proteins to identify novel medical applications resulting in a repurposing of known drugs. Despite these benefits of approved drugs, it is almost impossible to physically obtain a complete collection. Commercial vendors cover about 50-65% of drugs. Academic efforts such as the Johns Hopkins Clinical Compound Library (JHCCL) [1] and the NCGC Pharmaceutical collection [2] are only accessible via collaboration agreements. Thus, an affordable reference drug library obtainable for all screening centers is still missing. Here we report the generation of the CeMM Library of Unique Drugs (CLOUD), a focused collection of 314 systemically bioavailable small molecule drugs. The library was derived from FDA-approved drugs applying data mining and structural clustering techniques. Reduction of approved drugs to a set of systemically available small molecules and clustering based on their activity classes (e.g. dopamine receptor agonist) resulted in 244 compounds. In addition to this representative set of structural and biological space of drugs, CLOUD contains 35 drugs where the biological target is still unknown. Another 35 molecules representing the active forms of CLOUD prodrugs ensure the usability of the library for both biochemical and cell-based assays. Finally, all CLOUD drugs are delivered in a single 384-well plate in concentrations related to their therapeutic plasma levels to make high-throughput screening as comfortable as possible.
